# RISK ASSESSMENT FOR RADON EXPOSURE IN VARIOUS INDOOR ENVIRONMENTS

**DOI:** 10.1093/rpd/ncy284

**Published:** 2019-01-09

**Authors:** Jing Chen

**Affiliations:** Radiation Protection Bureau, Health Canada, 775 Brookfield Road, Ottawa, Ontario, Canada

## Abstract

Using data from a number of radon surveys, it was assessed that on average, radon progeny concentrations in Canadian homes are about three times higher than in school buildings, 4.7 times higher than in public buildings and indoor workplaces, and 12 times higher than in outdoor air. Canadian statistics show that most Canadians spend ~70% of their time indoors at home, 20% indoors away from home and 10% in outdoors. Due to relatively higher radon concentration in residential homes and longer time spent indoors at home, the exposure at home contributes to 90% of the radon-induced lung-cancer risk.

## INTRODUCTION

Radon (^222^Rn) is a naturally occurring radioactive gas generated by the decay of uranium-bearing minerals in rocks and soils. When radon gas escapes from the ground outdoors, it gets diluted and does not pose a health risk. However, in confined spaces, i.e. indoors, radon can accumulate to relatively high levels and become a health hazard when inhaled. In the air, radon decays further to its progenies or decay products. Radon gas contributes relatively little to the dose to the lung. It is the inhalation of the short-lived solid radon decay products and subsequent deposition on the walls of the airway epithelium of the bronchial tree that delivers most of the radiation dose to human lungs. Radon and its short-lived progenies in the atmosphere are the most important contributors to human exposure from natural sources of radiation, and have been identified as the second leading cause of lung cancer after tobacco smoking^(1–5)^.

Based on epidemiological studies on lung cancer risks associated with residential radon exposure^(6–8)^, the Government of Canada lowered the Canadian radon guideline for indoor environments from 800 to 200 Bq/m^3^ in June 2007^(9)^. This prompted a new wave of radon testing, with measurements carried out across Canada in homes, schools, offices and public buildings. In this study, average radon concentrations in different indoor environments are summarized based on published survey results and postings of project reports from local and federal governments and research organizations.

It is well known that the risk of radon-induced lung cancer increases with exposure to radon progeny and the duration of the exposure. Because radon is everywhere in varying concentrations, time-activity data are a key component of exposure assessment. In this study, radon gas concentrations are combined with time-location information and radon equilibrium factors to generate time- and location-weighted exposure estimates and to calculate associated contributions to the health risk from various indoor environments.

## RADON LEVELS IN VARIOUS ENVIRONMENTS

Radon is present everywhere in the air in varying concentrations. As demonstrated in the first cross-Canada residential radon survey in 19 cities in the 1970s, it is more likely to find high indoor radon concentrations in some geographic locations in Canada than others^(10)^. Since the first survey, many local governments, especially where elevated radon was observed, initiated radon surveys in residential homes, school and public buildings as well as various other indoor workplaces. Table 1 summarizes available data from a number of these surveys.

**Table 1.** Summary of radon concentrations in various indoor environments^(11–28)^.

City/region/province	Population (2016)	Building function	Number of places tested for radon	AM ± SD (Bq/m^3^)	Range (Bq/m^3^)	Reference #
Prince George	74 003	Homes	1436	185	(<DL, 1550)	11
		School buildings	44	30	(<DL, 240)	12
		Civic facilities	10	38	(<DL, 150)	13
		Federal buildings	14	25 ± 40	(<DL, 148)	14
Castlegar	8 039	Homes	158	373	(<50, 1250)	15
		School buildings	13	100	max. 855	12
Okanagan region	362 258	Homes	217	137 ± 163	(<DL, 1410)	16, 17
		Schools/daycares	131	44	(<DL, 400)	12
		Federal buildings	59	40 ± 51	(<DL, 276)	14
Calgary	1 392 609	Homes	2382	126	(<15, 3440)	18
		Homes	185	111 ± 92	(<DL, 850)	16, 17
		Provincial buildings	27	43	(<DL, 288)	19
		Federal buildings	20	19 ± 12	(<DL, 106)	14
Edmonton	1 321 426	Homes	170	101 ± 62	(<DL, 386)	16, 17
		Provincial buildings	10	24	(<DL, 58)	19
		Federal buildings	119	19 ± 14	(<DL, 169)	14
Ottawa	934 243	Homes	266	99 ± 116	(<DL, 1525)	16, 17, 20
		Federal buildings	255	30 ± 41	(<DL, 408)	14
Gaspesie region	90 311	Homes	174	158 ± 270	(<DL, 2923)	16
		School buildings	19	76 ± 116	(15, 663)	21
		Federal buildings	40	122 ± 251	(<DL, 1580)	14
Laurentides region	589 400	Homes	78	88 ± 124	(<DL, 757)	16
		School buildings	22	57 ± 65	(15, 453)	21
Outaouais region	382 604	Homes	62	111 ± 170	(<DL, 917)	16
		School buildings	24	44 ± 37	(15, 206)	21
Saskatchewan	1 098 352	Homes	1604	153 ± 132	(<DL, 2165)	16, 17
		School buildings	424	73 ± 91	(11, 1243)	22
		Provincial buildings	56	47 ± 32	(<DL, 120)	23
		Federal buildings	342	71 ± 91	(<DL, 530)	14
Nova Scotia	923 598	Homes	758	130 ± 247	(<DL, 2690)	16, 17, 24
		School buildings	377	62 ± 46	(<DL, 1312)	25
		Provincial buildings	20	27	(<DL, 200)	26
		Federal buildings	581	38 ± 69	(<DL, 870)	14
Prince Edward Island	142 907	Homes	113	46 ± 67	(<DL, 415)	16, 17
		Senior housings	38	43 ± 37	(11, 169)	27
		School buildings	46	68 ± 58	(10, 305)	27
		Public buildings	8	34 ± 46	(12, 146)	27
		Federal buildings	51	25 ± 31	(<DL, 204)	14
Yukon	35 874	Homes	225	175 ± 309	(<DL, 2360)	16
		School buildings	32	65 ± 58	(6, 430)	28
		Federal buildings	56	58 ± 53	(<DL, 290)	14
Canada^a^		Homes	7866	119	(<DL, 3440)	
		School buildings	1132	61	(<DL, 1312)	
		Public buildings	1668	38	(<DL, 1580)	

^a^Population weighted arithmetic mean (AM) from available data in this table.

A total of 7866 long-term measurement data were available for homes; the population weighted average radon concentration was 119 Bq/m^3^. Radon measurements were available for a total of 1132 school buildings; the average concentration was 61 Bq/m^3^, about half of radon concentration found in homes. Local civil facilities, provincial and federal buildings are all counted here as public buildings. Radon measurements were reported for a total of 1668 public buildings; the average radon concentration was 38 Bq/m^3^, only ~32% of that in residential homes.

Radon gas contributes relatively little to the dose to the lung. It is the inhalation of the short-lived solid radon decay products and subsequent deposition on the walls of the airway epithelium of the bronchial tree that delivers most of the radiation dose to human lungs. Health risk associated with radon exposure depends on radon progeny concentration, represented by the radon equilibrium equivalent concentration (EEC) which is estimated by measured radon gas concentration times the equilibrium factor *F*_eq_ (a factor describing the degree of disequilibrium between radon gas and its progeny). The characteristics of radon decay products differ significantly in different indoor environments. Many environmental factors as well as human activities and habits affect the value of *F*_eq_. Measured *F*_eq_ values vary widely from as low as 0.1 to as high as 0.8. Radon equilibrium factors in school and public buildings can differ significantly from those of residential dwellings. This is due primarily to the different indoor environmental conditions required or created by the activities that take place within the buildings, especially with respect to ventilation and aerosol particle concentrations. Based on a recent review on *F*_eq_ in various indoor environments^(29)^, we applied *F*_eq_*=* 0.6 to Canadian homes and *F*_eq_*=* 0.4 for school and other public buildings in this assessment.

The average radon progeny concentrations of 71.4 Bq/m^3^ (0.6 × 119 Bq/m^3^) in homes, 24.4 Bq/m^3^ (0.4 × 61 Bq/m^3^) in schools and 15.2 Bq/m^3^ (0.4 × 38 Bq/m^3^) in public buildings including indoor workplaces were used here to assess radon exposures in various indoor environments.

Radon also presents in outdoor air. However, measurements of outdoor radon levels are very limited in Canada. In this assessment, we take the worldwide averages of 10 Bq/m^3^ and *F*_eq_ = 0.6 for radon in outdoor air recommended by the UNSCEAR^(3)^. Radon progeny concentration of 6 Bq/m^3^ (0.6 × 10 Bq/m^3^) was used in the current assessment for all outdoor locations.

## TIME-ACTIVITY PATTERN

Time-activity data are a key component for population exposure assessment. Radon concentrations in different environments are combined with time-location information to generate time-weighted estimates for radon exposure. The General Social Survey: Canadians at work and home conducted by the Statistics Canada^(30, 31)^ provided updated time-activity data, representative of the Canadian population. The average daily time spent in major locations by age groups are summarized in Table 2.

**Table 2.** Daily time spent in major locations by age groups (daily percentages in bracket), mean EEC concentrations in different locations and associated annual exposures.

Age group	Location	Mean time spent (h)	Mean EEC (Bq/m^3^)	Annual Exposure (h Bq/m^3^)
Infants	Indoors at home	21.38 (89%)	71.4	557 184
(<1 y)	Indoors away from home	1.17 (5%)	15.2	6491
	Outdoors	0.95 (4%)	6	2081
	In vehicle	0.50 (2%)	6	1095
Young children	Indoors at home	17.73 (74%)	71.4	462 062
(1–4 y)	Indoors away from home	3.67 (15%)	71.4	95 644
	Outdoors	1.82 (8%)	6	3986
	In vehicle	0.78 (3%)	6	1708
Children	Indoors at home	17.12 (71%)	71.4	446 164
(5–11 y)	Indoors away from home	4.27 (18%)	24.4	38 029
	Outdoors	1.80 (8%)	6	3942
	In vehicle	0.81 (3%)	6	1774
Adolescents	Indoors at home	16.67 (69%)	71.4	434 437
(12–19 y)	Indoors away from home	4.98 (21%)	24.2	43 988
	Outdoors	1.48 (6%)	6	3241
	In vehicle	0.87 (4%)	6	1905
Adults	Indoors at home	16.03 (67%)	71.4	417 758
(20–59 y)	Indoors away from home	5.13 (21%)	15.2	28 461
	Outdoors	1.32 (6%)	6	2891
	In vehicle	1.52 (6%)	6	3329
Seniors	Indoors at home	18.63 (78%)	71.4	485 516
(60+ y)	Indoors away from home	2.98 (12%)	15.2	16 533
	Outdoors	1.32 (6%)	6	2891
	In vehicle	1.07 (4%)	6	2343

Generally speaking, Canadians spend ~21 h (90% of daily time) indoors. Young children and seniors spend more than 70% of their daily time indoors at home. On average, students spend ~5 h or 20% of the daily time indoors in school. Outdoor activities including time in vehicles only count for ~10% of the time.

## CONTRIBUTION ASSESSMENT FOR RADON EXPOSURE IN MAJOR LOCATIONS

Annual radon exposure can be estimated with knowledge of time-activity patterns, which specify where and how individuals spend their time, along with knowledge of radon activity concentrations in each microenvironment or physical space, as given in Table 2 for exposures in major locations by age groups. For infants, it is assumed that the time spent indoors other than home is most likely in civic facilities or public buildings where the mean of EEC is 15.2 Bq/m^3^. For young children, daycares are assumed to be the most common indoor locations outside the home. In Canada, the majority of child care is through unregulated arrangements, such as in the caregiver’s home. In this study, the EEC of 71.4 Bq/m^3^ is assigned to daycares. No information was found for radon progeny concentration in vehicles, so it is assumed that radon levels in a vehicle are the same as in outdoor air.

Table 3 provided total annual exposures for different age groups and percentage contributions by major locations. The total annual exposure depends on the amount of time spent indoors, especially indoors at home. It is highest for infants (98% of time at home), and then decreases with increased age until people become seniors, retired from work and spending more time at home. From the percentage contribution by location for the three major locations, one can see that exposure indoors contributes ~99% to the total annual exposure, and the majority comes from exposure at home. Exposure outdoors counts for only ~1% of the total annual exposure.

**Table 3.** Total annual exposures by age groups and contributions from exposure at various locations.

Age group	Total annual exposure (h Bq/m3)	% Contribution by location	Location
Infants	566 851	98.3%	Indoors at home
(<1 y)		1.1%	Indoors away from home
		0.6%	Outdoors + in vehicle
Young children	563 399	82.0%	Indoors at home
(1–4 y)		17.0%	Indoors away from home
		1.0%	Outdoors + in vehicle
Children	489 909	91.1%	Indoors at home
(5–11 y)		7.8%	Indoors away from home
		1.2%	Outdoors + in vehicle
Adolescents	483 572	89.8%	Indoors at home
(12–19 y)		9.1%	Indoors away from home
		1.1%	Outdoors + in vehicle
Adults	452 439	92.3%	Indoors at home
(20–59 y)		6.3%	Indoors away from home
		1.4%	Outdoors + in vehicle
Seniors	507 284	95.7%	Indoors at home
(60+ y)		3.3%	Indoors away from home
		1.0%	Outdoors + in vehicle

## RADON RISK ASSESSMENT

There are several radon risk models in the literature. In all risk models, lifetime risk of radon-induced lung cancer increases with increased exposure to radon progeny. The lifetime risk estimates could vary significantly between the various risk models considered. A previous study on variation range assessment for Canadian population risk of radon-induced lung cancer based on various risk models^(32)^ found that the lifetime risk estimates based on the EPA/BEIR-VI model^(33)^ agreed reasonably well with the averages of risk estimates from the five risk models considered in that study. In this study, the EPA/BEIR-VI radon risk model was used to assess radon risk. The mathematical form of the EPA/BEIR-VI model for the excess relative risk, *e*, is given as:

$$e\left(a\right)=\beta (W_{5-14}+0.78W_{15-24}+0.51W_{25+})\Phi_{age}\left(a\right)$$

where a is age in years. The parameter *β* (=0.0634) represents the increase of risk per unit exposure, expressed as excess relative risk per Working Level Month (WLM) (1 WLM = 6.37 × 10^5 ^h Bq/m^3^ EEC of radon). For a given radon exposure pattern, the cumulative exposure, *W* (expressed in WLM), can be calculated as the weighted summation of three time-since-exposure windows, namely *W*_5–14_ the exposure incurred between 5 and 14 y before age a; *W*_15–24_ the exposure incurred between 15 and 24 y before age a and *W*_25+_ the exposure incurred 25 y or more before age a. Exposure in the last 5 y is not biologically relevant to lung-cancer risk. Φ_age_ is a function decreasing with attained age.

The formulae for the calculation of lifetime risk of lung cancer are described in the BEIR IV report^(34)^. Briefly, the lifetime risk of lung cancer is given by the sum of the risks of lung-cancer death for each year *i*:

$$\begin{array}{c} R_{e}=\overset{110}{\underset{i=1}{\sum }}\frac{h_{i}(1+e_{i})}{h_{i}^{⁎}+h_{i}e_{i}}\prod_{k=1}^{i-1}\exp\left(-\left(h_{k}^{⁎}+h_{k}e_{k}\right)\right)\\ \cdot \left[1-\exp\left(-(h_{i}^{⁎}-h_{i}e_{i})\right)\right] \end{array}$$

where *R*_e_ is the absolute risk of lung cancer for a given exposure pattern; *h_i_* and h_i_* are the lung cancer and overall mortality rates for age *i*, respectively, and *e*_i_ is the excess relative risk due to exposure to radon progeny for age *i*. The lifetime probability of lung-cancer mortality is then the summation over years *i* from 1 to 110. A lifespan of 110 y is assumed. This study uses the Canadian age-specific mortality rates^(35)^ averaged over 5 y from 2008 to 2012.

**Figure 1.**
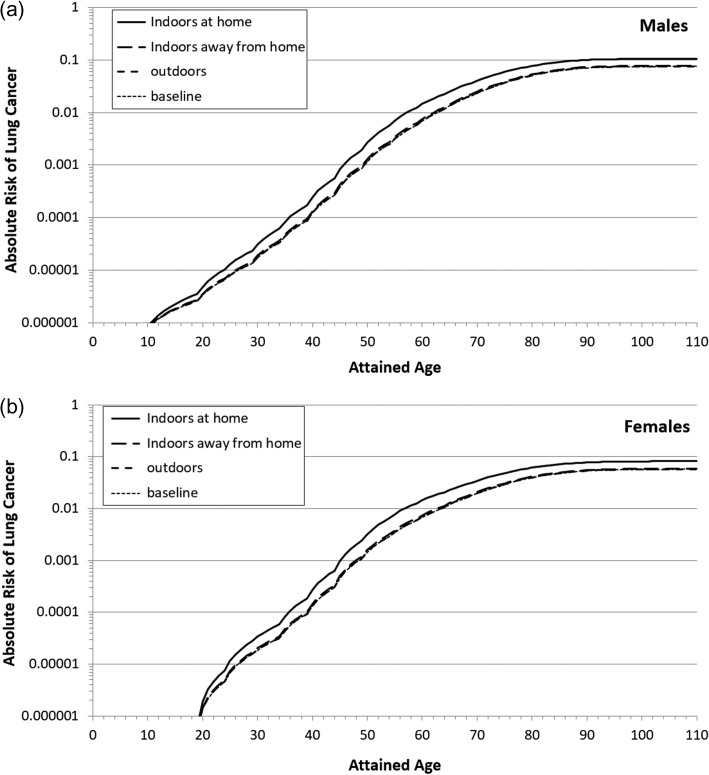
Absolute lung-cancer risk as a function of attained age for exposures at three major locations in comparison with baseline risk.

Lifetime risks were calculated for exposure patterns (as given in Table 3) during the lifetime at three major locations. The results are presented in Figure 1. Baseline risks, *R*_0_, were also calculated for comparison, they are risks when *e*_*i*_ = 0 in above equation.

The lung-cancer risks are zero for males under the age of 10 and for females under the age of 20. The lifetime baseline risks of lung cancer (i.e. by age of 110) are 0.075 for males and 0.057 for females. Exposures to radon anywhere increase the risk of developing lung cancer over the course of the lifetime. The results in Figure 1 showed that exposure at home contributes to the majority of the increased risk of lung cancer.

**Figure 2.**
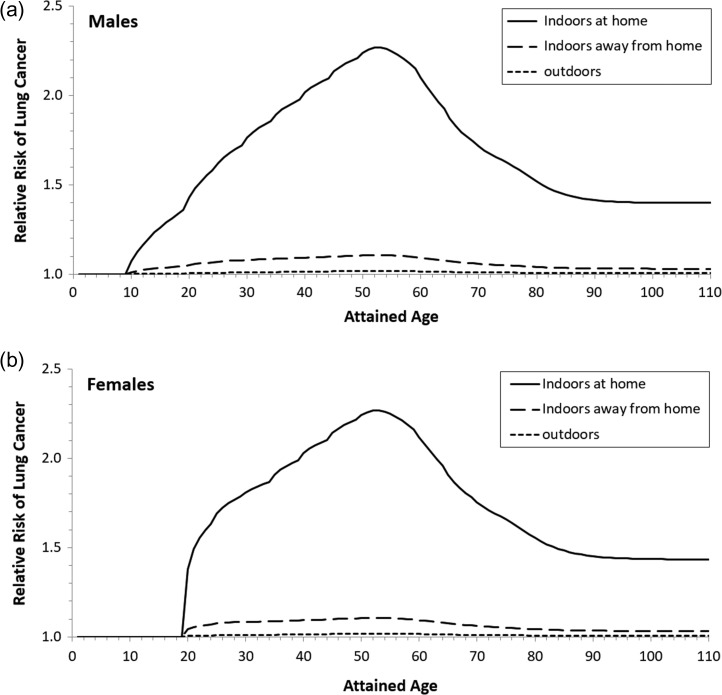
Relative risk of lung cancer as a function of attained age due to exposures at three major locations.

Relative risk (RR) is defined as RR = *R*_e_/*R*_0_, where *R*_0_ is the baseline risk. The RR describes the proportional increment in lung-cancer risk posed by radon exposure beyond the baseline. By converting the results of absolute risks in Figure 1 into relative risks, we can more clearly see the significant contributions of exposure at home to radon-induced lung cancer, as demonstrated in Figure 2.

Even though lung cancer was not observed among young children, their exposures to relatively higher radon concentrations at home contribute to significant increases in relative risks later in their life. This was clearly demonstrated for females, where a rapid increase in relative risks around age 20 can be seen. Figure 2 also indicates that exposure to radon at average Canadian levels adds considerably to the risk of lung cancer for all age groups, and that the relative risk for people exposed increases steadily from early adulthood to late middle age.

Risks of radon-induced lung cancer relative to the baseline risk can vary over the lifetime depending on varying exposure pattern and other environmental factors. Regardless the risk values, risks due to exposure at home always contribute to ~90% of the risk due to total exposure at all locations.

## CONCLUSIONS

The risk of radon-induced lung cancer increases with exposure to radon progeny and the duration of the exposure. On average, radon progeny concentrations in Canadian homes are about three times higher than in school buildings, 4.7 times higher than in public buildings and indoor workplaces, and 12 times higher than in outdoor air. Canadian statistics showed that most Canadians spend ~70% of their time indoors at home, 20% indoors away from home and 10% in outdoors.

Due to relatively higher radon concentration in residential homes and longer time spent indoors at home, exposure at home contributes to 90% of the radon-induced lung-cancer risk. The assessment results clearly indicate that reducing radon exposure in residential homes is the most important and most effective way for prevention of radon-induced lung cancer.
